# T Cell-Derived IL-17A Induces Vascular Dysfunction via Perivascular Fibrosis Formation and Dysregulation of ^·^NO/cGMP Signaling

**DOI:** 10.1155/2019/6721531

**Published:** 2019-07-18

**Authors:** Rebecca Schüler, Panagiotis Efentakis, Johannes Wild, Jérémy Lagrange, Venkata Garlapati, Michael Molitor, Sabine Kossmann, Matthias Oelze, Paul Stamm, Huige Li, Katrin Schäfer, Thomas Münzel, Andreas Daiber, Ari Waisman, Philip Wenzel, Susanne Helena Karbach

**Affiliations:** ^1^Center for Thrombosis and Hemostasis (CTH), University Medical Center Mainz, Mainz, Germany; ^2^Institute for Molecular Medicine, University Medical Center Mainz, Mainz, Germany; ^3^Center for Cardiology-Cardiology I, University Medical Center Mainz, Mainz, Germany; ^4^German Center for Cardiovascular Research (DZHK)-Partner Site Rhine-Main, Germany; ^5^Department of Pharmacology, University Medical Center Mainz, Mainz, Germany

## Abstract

**Aims:**

The neutrophil recruiting cytokine Interleukin-17A (IL-17A) is a key component in vascular dysfunction and arterial hypertension. Moreover, IL-17A has a central role for the vascular infiltration of myeloid cells into the arterial wall in Angiotensin II-induced vascular inflammation. The intention of our study was to analyze the impact of T cell-derived IL-17A on hypertension, vascular function, and inflammation.

**Methods and Results:**

Chronic IL-17A overexpression in T cells (CD4-IL-17A^ind/+^ mice) resulted in elevated reactive oxygen species in the peripheral blood and a significant vascular dysfunction compared to control mice. The vascular dysfunction seen in the CD4-IL-17A^ind/+^ mice was only accompanied by a modest and nonsignificant accumulation of inflammatory cells within the vessel wall. Therefore, infiltrating myeloid cells did not serve as an explanation of the vascular dysfunction seen in a chronic IL-17A-driven mouse model. In addition to vascular dysfunction, CD4-IL-17A^ind/+^ mice displayed vascular fibrosis with highly proliferative fibroblasts. This fibroblast proliferation was induced by exposure to IL-17A as confirmed by *in vitro* experiments with primary murine fibroblastic cells. We also found that the ^·^NO/cGMP pathway was downregulated in the vasculature of the CD4-IL-17A^ind/+^ mice, while levels of protein tyrosine kinase 2 (PYK2), an oxidative stress-triggered process associated with T cell activation, were upregulated in the perivascular fat tissue (PVAT).

**Conclusions:**

Our data demonstrate that T cell-derived IL-17A elicits vascular dysfunction by mediating proliferation of fibroblasts and subsequent vascular fibrosis associated with PYK2 upregulation.

## 1. Introduction

Angiotensin II- (AngII-) induced vascular dysfunction is mediated by the invasion of inflammatory leukocytes, including T cells and myelomonocytic cells, into the vasculature [1, 2]. The infiltrating leukocytes represent one source of reactive oxygen and nitrogen species (ROS/RNS). The resulting imbalance of pro- and antioxidative molecules drives vascular dysfunction and arterial hypertension [1–3]. Vascular inflammation in AngII-induced hypertension and vascular dysfunction was described to be IL-17A-dependent [4, 5]. IL-17A derives from *γδ* T cells [6] and CD4^+^ T helper cells (Th17 cells) [7] and recruits and activates myeloid cells [8, 9]. IL-17A deficiency dampened vascular dysfunction, arterial hypertension, and vascular inflammation after four weeks of AngII infusion [4, 5]. In atherogenesis, the contribution of IL-17A is still elusive and data are partly contradictory: while IL-17A inhibition reduced the atherosclerotic lesion size in ApoE^−/−^ mice [10], T cell-specific deficiency of *suppressor of cytokine signaling-3* (SOCS3) led to increased IL-17 levels, which were associated with a smaller atherosclerotic lesion size compared to those of control mice [11]. Thus, the adequate levels of IL-17A and the correlating and complement cytokines might be more important for vascular homeostasis than absolute IL-17A levels.

The autoimmune skin disease psoriasis—which is the most prevalent IL-17A-dependent disease—is also correlated with an increased cardiovascular burden [12, 13]. Here again, IL-17A is one key player in connecting skin and vascular diseases [14, 15]. We have recently shown that vascular dysfunction in murine psoriasis is correlated with peripheral IL-17A levels and neutrophil infiltration into the aortic vessel wall and with the severity of skin disease. Successful anti-IL-17A treatment of psoriatic skin lesions diminished peripheral oxidative stress levels, proinflammatory cytokines, and vascular inflammation [16]. Interestingly, aortic neutrophil infiltration in murine psoriasis was lower than that in mice with AngII-induced vascular dysfunction [1] although they exhibited a comparable vascular dysfunction [16]. Therefore, we hypothesized that either the long-term vascular inflammation or additional effects of IL-17A other than neutrophil infiltration contribute to the impaired vascular function in the murine model of psoriasis [16]. Several studies describe direct effects of IL-17A on endothelial NO and ROS/RNS production: acute IL-17A treatment was described to increase the inhibitory eNOS Thr495 phosphorylation in endothelial cells, decreased NO-dependent relaxation responses in isolated mouse aorta [17], and IL-17A-induced ROS/RNS formation in endothelial cells of the blood-brain barrier [18]. Moreover, IL-17A contributed to aortic stiffening and to vascular fibrosis [19]—processes that are relevant in vascular dysfunction and a characteristic feature of arterial hypertension.

Within our study, we analyzed vascular function, inflammation, and oxidative stress formation in a mouse model of T cell-dependent IL-17A overexpression. We found a direct influence of IL-17A on oxidative stress formation, vascular fibrosis, and the ^·^NO/cGMP signaling resulting in severe vascular dysfunction without direct vascular immune cell infiltration.

## 2. Material and Methods

### 2.1. Mice

CD4-IL-17A^ind/+^ mice resulting from crossing the CD4-Cre [20] mice with the IL-17A^ind/ind^ mice [15, 21] were used for experiments. Cre-mediated recombination leads to overexpression of IL-17A combined with the green fluorescent protein (GFP) expression in CD4^+^ and CD8^+^ T cells. As controls, Cre-negative IL-17A^ind/+^ mice were used, which do not show any phenotype alterations and which are comparable to wild-type mice. For the experiments, we used 12-14-week-old male mice, all with the C57BL/6J background. Animals were housed in the animal facility of the University Medical Center Mainz TARC (Translational Animal Research Center) in accordance with FELASA guidelines and approved by the TARC and the Animal Care and Use Committee (IACUC) from the Land of Rhineland-Palatine (RLP) (approval numbers: 23 177-07/G 10-1-019 and G 15-1-051).

### 2.2. Bio-Plex Assay

Different cytokine concentrations in plasma were detected using the Bio-Plex Pro™ Mouse Cytokine Grp I Panel 23-Plex (Bio-Rad) following the manufacturer's description. The plate was read using the Luminex MAGPIX® instrument.

### 2.3. Aortic Relaxation Studies

Isolated 4 mm aortic sections were mounted in organ chambers, and endothelium-dependent vascular relaxation in response to acetylcholine (ACh), smooth muscle cell-dependent vascular relaxation in response to glyceryl trinitrate (GTN), and aortic contraction in response to phenylephrine (Phe) were measured as described previously [22].

### 2.4. Echocardiography

Mice were anesthetized in a chamber containing 2-4% isoflurane mixed with 0.2 l/min 100% O_2_, and during measurements, narcosis was retained using 1-2% isoflurane with 0.2 l/min 100% O_2_. Mice were fixed on a heated table on a rail system (VisualSonics, Canada). Ultrasound measurement was conducted using the Vevo 770 or 3100 System (VisualSonics) with a 400 MHz mouse transducer.

### 2.5. Blood Pressure Measurements

Blood pressure was recorded via carotid catheters. Mice were anesthetized for catheter implantation with intraperitoneal injection of midazolam (5 mg per kg body weight; Ratiopharm GmbH), medetomidine (0.5 mg per kg body weight), and fentanyl (0.05 mg per kg body weight; Janssen-Cilag GmbH). To antagonize anesthesia after the surgery, mice were injected subcutaneously with atipamezole (0.05 mg per kg body weight) and flumazenil (0.01 mg per kg body weight). Mice recovered 1 week from surgery before measurement was recorded using receiver platforms and the Dataquest system (DSI).

### 2.6. Flow Cytometric Analysis

Single cell solutions of the spleen, kidneys, aorta, and perivascular adipose tissue (PVAT) were prepared to be stained with fluorophore-coupled antibodies for flow cytometric analyses. After blocking unspecific binding with Fc-block (Bio X Cell, USA), the following monoclonal antibodies were used for surface staining: CD45.2 (clone: 104, APC-eFluor780, eBioscience), CD90.2 (clone: 53-2.1, APC-eFluor780, eBioscience or PerCP, BioLegend), CD3 (clone: 145-2C11, PerCP, BD Biosciences), CD4 (clone: GK1.5, FITC, BioLegend or clone: RM4-5, V500, BD Biosciences), CD8 (clone: 53-6.7, PE-Cy7, eBioscience), CD11b (clone: M1/70, PE-Cy7, eBioscience), F4/80 (clone: BM8, APC, BioLegend), Ly6G (clone: 1A8, PE, BioLegend), and Ly6C (clone: AL-21, BD Biosciences).

Samples were acquired using the FACSCanto™ II (BD). Analysis was performed using the FlowJo Software (BD, USA).

### 2.7. ROS/RNS Measurement

Reactive oxygen and nitrogen species (ROS/RNS) were measured in whole blood or in isolated CD11b^+^ cells with L-012-enhanced chemiluminescence. CD11b^+^ splenocytes were isolated using CD11b MicroBeads (MACS, Miltenyi Biotec) according to the manufacturer's protocol. To stimulate the oxidative burst of leukocytes, whole blood or isolated CD11b^+^ cells were incubated for 10 or 20 minutes with phorbol 12,13-dibutyrate (PDBu) as described previously [23, 24]. To measure oxidative stress in vascular tissue, thoracic aortic sections were incubated either with buffer containing protease inhibitors or in addition with 500 *μ*M eNOS-inhibitor *N*_*ω*_-nitro-L-arginine methyl ester hydrochloride (L-NAME) for 30 min. After incubation, aortic rings were embedded in medium and snap-frozen in liquid nitrogen. Cryosections of aortas (8 *μ*m) were incubated with the superoxide-sensitive dye dihydroethidium (DHE, 1 *μ*M) to perform fluorescence oxidative microtopography. In the presence of ROS, a red (2-hydroxy)ethidium fluorescence (extinction: 535 nm, emission 610 nm) is visible, and in green, autofluorescence of the lamina was visible [1].

### 2.8. Histology

Aortic rings were fixed in 4% zinc formalin for 24 hours and embedded in paraffin at the histology core facility of the University Medical Center Mainz. A combined Masson's trichrome–Verhoeff's elastica (MTC-VES) staining was performed on 3 *μ*M thick cross-sections to simultaneously visualize elastic fibers (black) and fibrosis (blue). Fibrin appears red, fibrosis blue, and cell nuclei black. The adventitia and aortic wall thickness in histology were measured with ImageJ software. Sections were stained with Sirius red and examined under polarized light to visualize interstitial collagen fibers. Aortic wall thickness and thickness of the collagen-positive area in the adventitia were quantified.

Aortic rings and aortic sections were immunostained with antibodies against *α*-smooth muscle cell actin (*α*SMa, Abcam), fibroblast-specific protein 1 (FSP1, Novus Biologicals), CD31 (Dianova), and DAPI (Invitrogen).

### 2.9. Aortic Ring Sprouting Assay

For this assay, an aortic ring of 0.5 mm was cut after PVAT was removed. Rings were placed onto Matrigel in 24-well plates and incubated in the commercially available Endothelial Cell Growth Medium 2 Kit (PromoCell, Heidelberg, Germany). Images of the aortic rings were taken after 4 days in culture at 5x magnification. The whole protocol was adapted from Baker et al. [25].

### 2.10. Cell Culture Experiments

Primary mouse fibroblasts or vascular smooth muscle cells (VSMCs) were used for cell culture experiments. Fibroblasts were cultured at 37°C, 5% CO_2_ in Dulbecco's modified Eagle's medium (DMEN) containing 1% (*v*/*v*) FCS. Cells were plated at a density of 1000 cells/well in 96-well plates 24 h prior to treatment. Afterwards, cells were starved for 3 h, and different concentrations of IL-17A (0 ng/ml, 0.2 ng/ml, 100 ng/ml, and 200 ng/ml IL-17A) were added in serum-free medium, followed by an incubation time of 48 h. Subsequently, cells were harvested for MTT proliferation assay.

### 2.11. MTT Proliferation Assay

Treated fibroblasts and VSMCs were incubated with MTT solution (3-(4,5-dimethyl-2-thiazolyl)-2,5-diphenyl-2H-tetrazolium bromide) (M-5655, Sigma-Aldrich, St. Louis, MO, USA), directly added to the medium (final concentration 0.5 mg/ml) for 4 h at 37°C. Viable proliferating cells take up the MTT solution and reduce it, resulting in a purple color change of the solution. Medium was removed after 4 h, and cells were solubilized with dimethyl sulfoxide (DMSO). Absorbance of the purple color complex was measured with the Tecan microplate spectrophotometer (Tecan Spark, Tecan Inc., Männedorf, Switzerland) at 540 nm. Results of the treated cells were normalized to the optical density (OD) of controls.

### 2.12. ^·^NO Detection via Electron Spin Resonance Spectroscopy

To detect ^·^NO produced by endothelial cells in aortas, aortic rings of 4 mm were stimulated with 10 *μ*M calcium ionophore and incubated with Fe(diethyldithiocarbamate)_2_ (Fe(DETC)_2_) solution (0.2 mmol/l in PBS Ca^2+^/Mg^2+^) at 37°C, 10% CO_2_ for one hour and stimulated with calcium ionophore A23187 (10 *μ*M) for 1 h. After snap freezing, aortic samples were kept in liquid nitrogen. ^·^NO generation was measured with electron paramagnetic resonance- (EPR-) based spin trapping with the Fe(DETC)_2_ colloid with a MiniScope MS400 table-top X-band spectrometer (Magnettech, Germany) [26, 27].

### 2.13. eNOS *S*-Glutathionylation

M-280 sheep anti-mouse IgG-coated beads (Invitrogen) were used along with a monoclonal mouse eNOS antibody (BD Transduction Laboratory) as described previously [28]. Beads were loaded with the eNOS antibody and cross-linked according to the manufacturer's instructions. Aortic homogenates were incubated with the eNOS antibody beads, precipitated with a magnet, washed, transferred to the gel, and subjected to SDS-PAGE. Then, a standard Western blot procedure with a monoclonal mouse antibody against S-glutathionylated proteins from ViroGen (Watertown, MA) (dilution of 1 : 1000) under nonreducing conditions was performed. Signal disappearance upon incubation with 2-mercaptoethanol served as a control. After stripping of the membrane, the bands were stained for eNOS to allow normalization of the signals.

### 2.14. Western Blot Analysis

Aortic or PVAT samples were pulverized in liquid nitrogen and partially pooled together. Subsequently, pulverized tissues were lysed with RIPA buffer (Sigma-Aldrich) supplemented with protease-phosphatase inhibitor cocktail (Thermo Scientific), and protein was determined using the Lowry assay. According to their protein content, lysates were normalized to the same final protein concentration and mixed with a Laemmli lysis buffer to generate Western blot samples. Samples were electrophoresed on SDS-PAGE gels (7.5-12%) and transferred on 0.45 *μ*m Polyvinylidenfluorid (PVDF) membranes. PVDF membranes were blocked using 3% BSA and incubated with the following primary antibodies overnight at 4°C: p-eNOS (BD), eNOS, alpha-actinin (Cell Signaling Technology), pVASP S239 (Millipore), sGCa1 and sGCb1 (Abcam), PYK2 (Cell Signaling), and GCH-1 (Abnova). Primary antibodies were used at a dilution range of 1 : 500-1 : 2000. After the incubation, the appropriate anti-mouse and/or anti-rabbit (Cell Signaling Technology) secondary antibodies (1 : 2000) were used. Membranes were exposed using appropriate ECL Western Blotting Substrate (Thermo Scientific) and imaged on an automated imaging equipment (FUSION CCD Imager). Densitometrical analysis was performed using the appropriate software. Results were presented as relative integrated optical density.

### 2.15. RNA Isolation and Quantitative Real-Time PCR

Aortic tissue was lysed and RNA was isolated using TRIzol® (Thermo Fisher) according to their description. RNA concentration was measured with the NanoDrop spectrophotometer (Thermo Fisher). 0.5 *μ*g of total RNA was used for quantitative real-time PCR (qPCR) using the TaqMan® Gene Expression Assay (Applied Biosystems™) according to the manufacturer's protocol with the following primers: *Vcam1*, *Cybb*, *Nos3*, *Mmp2*, *Mmp9*, and *Tgfb*. The comparative delta CT method was used for relative mRNA quantification [29]. We normalized gene expression to the endogenous control (*TBP* mRNA), and the expression of the target gene mRNA of each sample was expressed relative to that of the control.

### 2.16. Statistical Analysis

Statistical analysis was performed with GraphPad Prism® Software. For two groups, Student's unpaired *t*-test was used; for more than two compared groups, one-way ANOVA, with Bonferroni's multiple comparison post-hoc test was used if normal distribution was given. In the case of no normal distribution, Mann–Whitney *t*-test or Kruskal-Wallis test (with Dunn's multiple comparison post-hoc test) was applied accordingly. (Data was analyzed for normal distribution with the Kolmogorov–Smirnov test.) Aortic relaxation curves were analyzed by two-way analysis of variance with the Bonferroni post hoc test. Cell culture experiments were analyzed with a one-way ANOVA test. *P* values of <0.001, <0.01, and <0.05 were considered statistically significant and marked by 3, 2, and 1 asterisks (^∗^), respectively. Data are presented as mean ± SEM.

## 3. Results

### 3.1. Mice Overexpressing IL-17A in T Cells Display a Significant Endothelial Dysfunction

**Figure 1:**
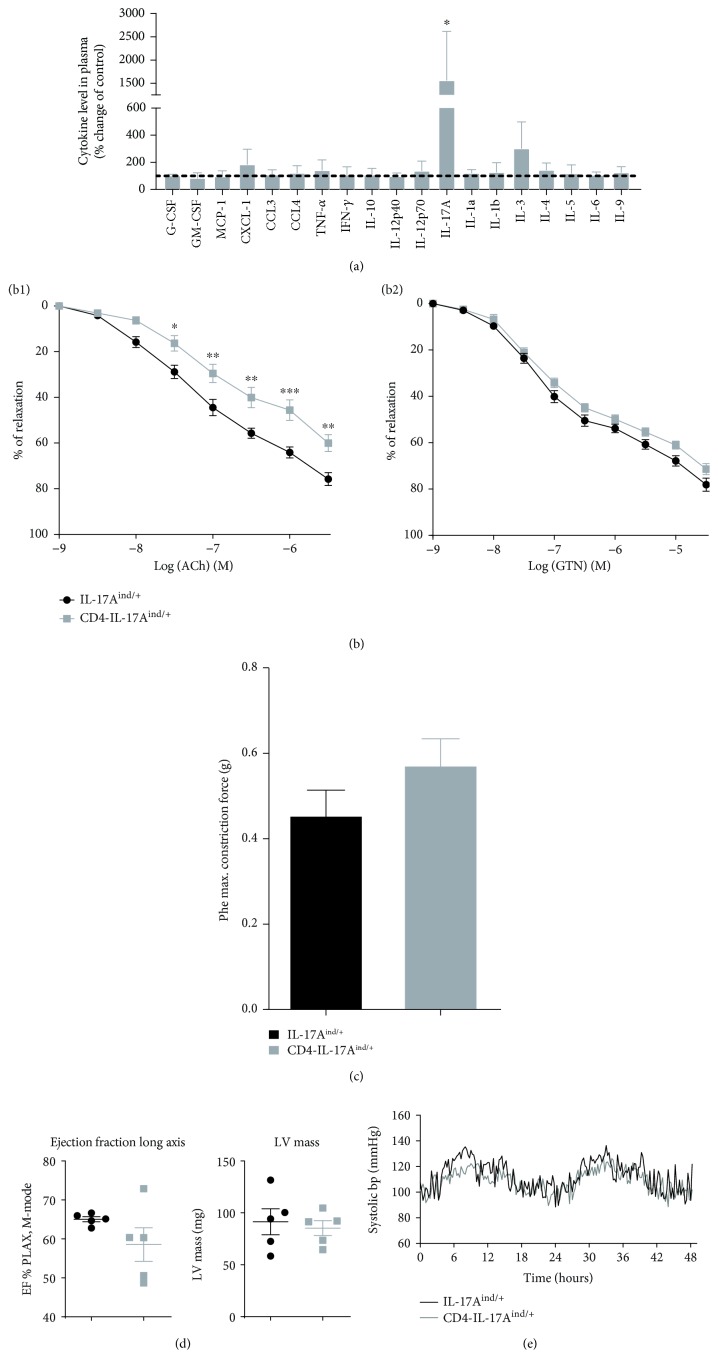
Overexpression of IL-17A in T cells results in vascular dysfunction per se. (a) Cytokine profile of CD4-IL-17A^ind/+^ mice in plasma measured with a Bio-Plex assay kit. IL-17A^ind/+^ control mice were normalized to 100% (indicated with the dashed line); percentual change compared to control is shown for CD4-IL-17A^ind/+^ mice; *n* = 3–6; either Student's unpaired *t*-test or Mann–Whitney *t*-test. (b) Cumulative concentration relaxation curves of aortic rings of CD4-IL-17A^ind/+^ and IL-17A^ind/+^ control mice in response to ACh (b1) and GTN (b2); *n* = 12–15; two-way ANOVA with Bonferroni post hoc test. (c) Maximal aortic constriction in response to a phenylephrine bolus in organ chamber from CD4-IL-17A^ind/+^ and IL-17A^ind/+^ control mice; *n* = 13; Student's unpaired *t*-test. (d) Cardiac function measured via echocardiography; *n* = 5; Student's unpaired *t*-test. (e) Systolic blood pressure detection via carotid artery telemetry catheter implantation of mice over 48 hours; *n* = 3–5. Data (a–d) are presented as mean ± SEM.

As CD4-IL-17A^ind/+^ mice constitutively overexpress IL-17A in T cells, they serve as a model of overabundance of Th17 cells [21]. We verified the T cell-specific IL-17A expression by GFP expression in CD3^+^ T cells in spleen and aorta (Figure S1A and S1B), as GFP expression is associated with that of the transgenic IL-17A [21]. CD4-IL-17A^ind/+^ mice developed normally, had normal outer appearance and behaviour, and showed no differences in survival compared to control mice (data not shown). The cytokine profile in the CD4-IL-17A^ind/+^ plasma confirmed significantly higher levels of only IL-17A compared to that in the control mice, whereas other cytokine concentrations remained unaltered (Figure 1(a)). We conclude that the CD4-IL-17A^ind/+^ mice constitute a unique murine model for studying the chronic effect of T cell-derived IL-17A on vascular function without additional cytokine alteration as confounders.

When we analyzed the response of the aorta rings of the CD4-IL-17A^ind/+^ mice, we found a significantly impaired vasorelaxation in response to acetylcholine (ACh) in comparison to IL-17A^ind/+^ control mice (Figure 1(b), b1). In contrast, there was no change in aortic relaxation in response to the endothelium-independent vasodilator glyceryl trinitrate (GTN) in CD4-IL-17A^ind/+^ mice (Figure 1(b), b2), and phenylephrine-induced aortic constriction was not significantly altered either, although it increased by trend (Figure 1(c)). Endothelial dysfunction in CD4-IL-17A^ind/+^ mice was not accompanied by a change in cardiac function or an increase in blood pressure (Figures 1(d) and 1(e)).

### 3.2. T Cell-Specific IL-17A Expression Does Not Lead to an Infiltration of Myeloid Cells into the Aortic Vessel Wall

**Figure 2:**
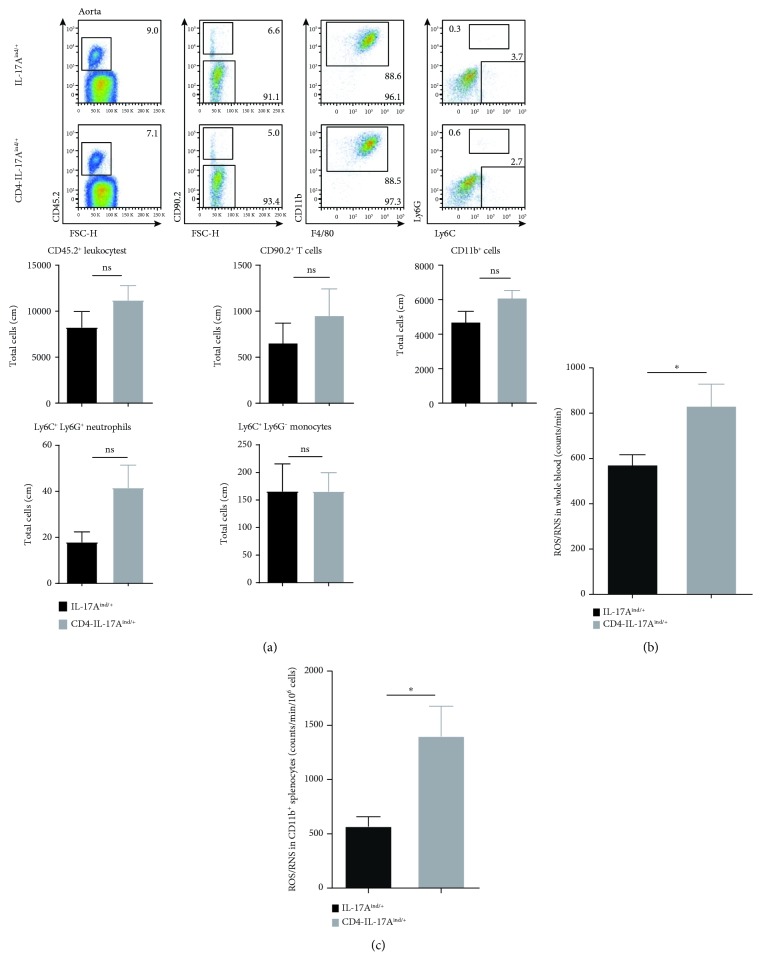
T cell-specific IL-17A overexpression does not result in an infiltration of inflammatory cells into the aortic wall; nevertheless, CD11b^+^ cells are more reactive. (a) Flow cytometric analysis of aortas from CD4-IL-17A^ind/+^ compared to IL-17A^ind/+^ control mice. Dot plot shows gating strategy of aortic samples. Cells were pregated on living cells and gated on CD45.2^+^, CD90.2^−^, CD11b^+^, F4/80^+^, and either Ly6G^+^Ly6C^+^ neutrophils or Ly6C^single+^ monocytes. Quantitative analysis of aortic flow cytometric analysis; *n* = 3–7 mice per group; either Student's unpaired *t*-test or Mann–Whitney *t*-test. (b) ROS/RNS measurement in whole peripheral blood was performed after stimulation with PDBu for 20 minutes; repeated measurements of pooled samples; *n* = 11–12 mice per group; unpaired Student's *t*-test. (c) ROS/RNS detection in isolated CD11b^+^ splenocytes after 20 min stimulation with PDBu; *n* = 5; Student's unpaired *t*-test. Data are presented as mean ± SEM.

As IL-17A was shown to modulate the recruitment of neutrophils during inflammation [9, 30], we expected vascular immune cell infiltration to the underlining reason for the observed endothelial dysfunction. Interestingly, impaired vascular function triggered by T cell-derived IL-17A was associated only with a moderate accumulation of CD11b^+^ myelomonocytic cells, Ly6G^+^Ly6C^+^ neutrophils, or Ly6G^−^Ly6C^+^ monocytes into the aortic vessel wall (Figure 2(a)). Total aortic CD45^+^ inflammatory cells and T cells were not significantly increased either (Figure 2(a)). Likewise, the myeloid cell compartment in peripheral blood was not significantly different from that of control animals (data not shown). In line with the vessel wall findings, the perivascular adipose tissue (PVAT) of CD4-IL-17A^ind/+^ mice only revealed a slight but not-significant increase in CD11b^+^ myeloid cells and Ly6G^+^Ly6C^+^ neutrophils compared to control mice (Figure S2A). Furthermore, no renal inflammation was detectable in CD4-IL-17A^ind/+^ mice despite the increased levels of T cell-derived IL-17A in peripheral blood (Figure S2B).

In contrast, we detected increased ROS/RNS levels in the peripheral blood of the CD4-IL-17A^ind/+^ mice as compared to those of the control animals (Figure 2(b)). Moreover, isolated CD11b^+^ cells of CD4-IL-17A^ind/+^ mouse spleens showed an augmented ROS/RNS production compared to those of the control mice (Figure 2(c)). These data suggest that overexpression of IL-17A alone leads to increased peripheral oxidative stress formation and compromises vascular function.

**Figure 3:**
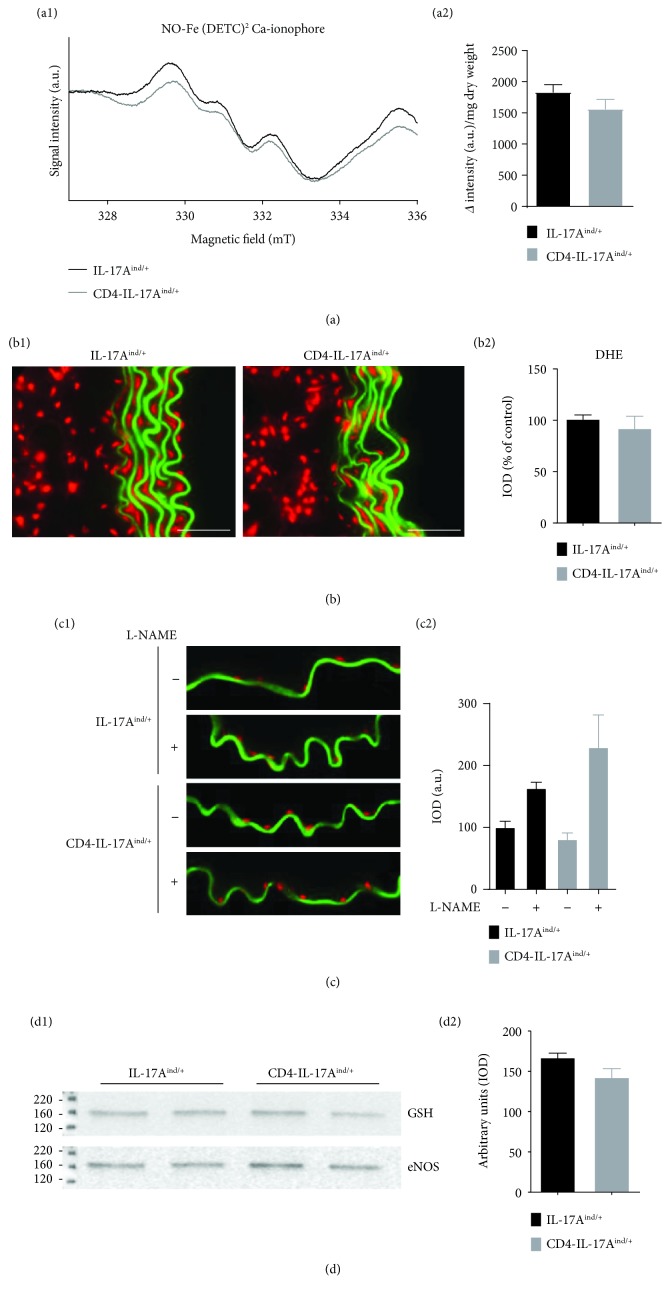
No eNOS uncoupling in CD4-IL-17A^ind/+^ mice. (a) NO production in aortic tissue was measured via electron paramagnetic resonance- (EPR-) based spin trapping. a2 shows the quantification of the delta intensity normalized to dry weight of samples; *n* = 9–10; Student's unpaired *t*-test. (b) Oxidative fluorescence microtopography of aortic sections of CD4-IL-17A^ind/+^ and IL-17A^ind/+^ mice. b1: representative picture of isolated aortic sections stained with dihydroethidium (DHE, 1 *μ*M). Autofluorescence of the laminae is visible in green, superoxide formation in red fluorescence; scale bar = 50 *μ*m. b2: quantification of superoxide formation in the aortas is shown as a percentage of control; *n* = 6–7; unpaired Student's *t*-test. (c) ROS formation in aortic tissue (endothelial scan) of CD4-IL-17A^ind/+^ and IL-17A^ind/+^ mice, incubated with L-NAME (eNOS inhibitor) or buffer. c1: representative DHE photomicrotopographs of aortic cryosections with focus on the endothelial layer; ROS formation appears in red. c2: quantification; *n* = 3; comparison of IL-17A^ind/+^ control mice with CD4-IL-17A^ind/+^ mice or comparison of IL-17A^ind/+^+L-NAME mice with CD4-IL-17A^ind/+^+L-NAME mice; Mann–Whitney *t*-test. (d) *S*-Glutathionylation (GSH) of eNOS was determined by eNOS immunoprecipitation from aortic protein samples of CD4-IL-17A^ind/+^ and IL-17A^ind/+^ mice, followed by antiglutathione staining and normalization to eNOS. Disappearance of the antiglutathione staining in the presence of 2-mercaptoethanol (*2-Me*) served as a control. d1: representative original blot. d2: densitometric analysis; *n* = 4 (per *n*, 3-4 aortas were pooled); Mann–Whitney *t*-test. Data are shown as mean ± SEM.

In contrast to the increased ROS/RNS levels, we detected little change in the levels of NO in the aortas of CD4-IL-17A^ind/+^ mice (Figure 3(a)) and we did not detect any changes in aortic superoxide formation in CD4-IL-17A^ind/+^ mice (Figure 3(b)). Moreover, incubation of aortic rings with the eNOS inhibitor L-NAME increased superoxide formation in the endothelial layer of aortic sections of CD4-IL-17A^ind/+^ aortas to the same extent as in the control mice (Figure 3(c)). In agreement, we found no *S*-glutathionylation of eNOS detectable in CD4-IL-17A^ind/+^ aortas (Figure 3(d)). These findings indicate that chronic IL-17A overexpression does not induce eNOS uncoupling in CD4-IL-17A^ind/+^ mice.

### 3.3. Vascular Dysfunction in Mice with IL-17A Overexpressing T Cells Is Characterized by Increased Vascular Fibrosis

**Figure 4:**
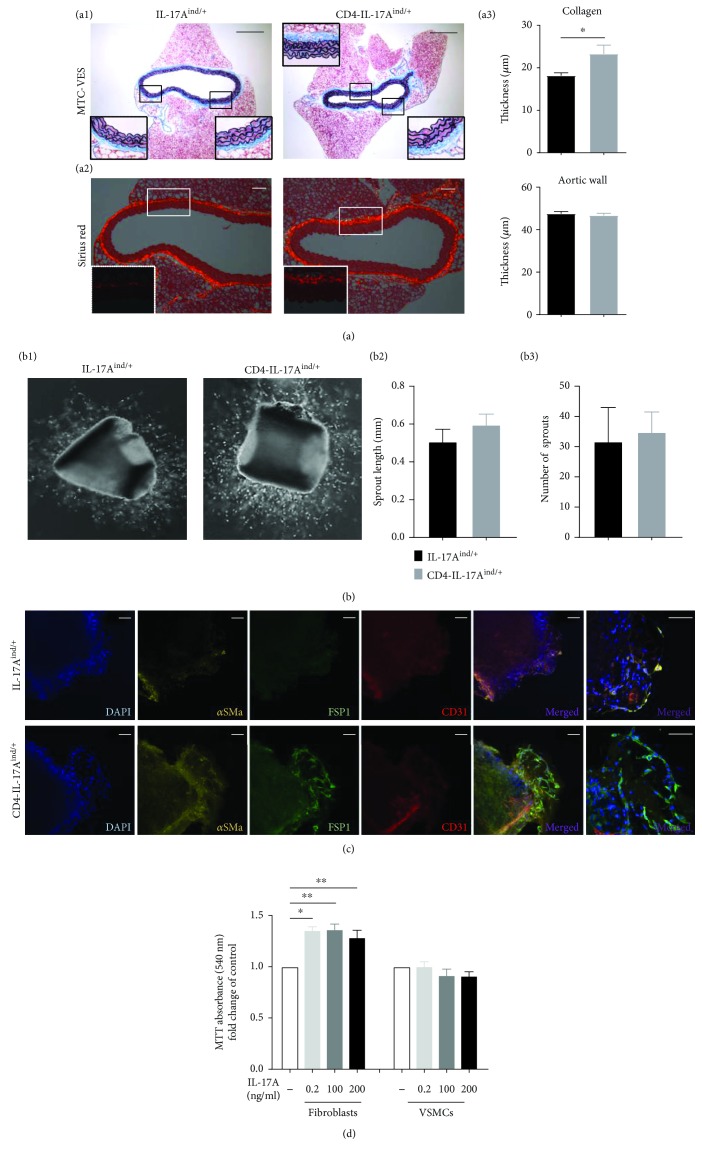
IL-17A induced perivascular fibrosis in CD4-IL-17A^ind/+^ mice and increased fibroblast migration and proliferation. (a) Combined Masson's trichrome–Verhoeff's elastica (MTC-VES) staining (a1) and Sirius red staining (a2) of aortic rings of CD4-IL-17A^ind/+^ and control mice. Representative pictures are shown; scale bar for both stainings = 100 *μ*m. a3: quantification of aortic wall thickness and thickness of collagen-positive area in adventitia are shown. Thickness was measured at 10 different points per section with ImageJ software; *n* = 6; unpaired Student's *t*-test. (b) Aortic sprouting assay of CD4-IL-17A^ind/+^ and control mice. b1: representative image of aortic rings on Matrigel covered plates at 5x magnification. Quantification of sprout length (b2) and number of sprouts (b3); *n* = 2–3 mice. (c) Aortic ring staining after sprouting assay of CD4-IL-17A^ind/+^ and control mice. Aortic rings were stained for DAPI (blue), *α*-smooth muscle cell actin (*α*SMa, yellow), fibroblast-specific protein 1 (FSP1, green), and CD31 (red); scale bar = 60 *μ*m. A representative image per group is shown; *n* = 2–3 mice per group. (d) MTT analysis of fibroblasts and smooth muscle cells (SMCs) cultivated with different concentrations of IL-17A; *n* = 3; one-way ANOVA with Bonferroni's post-hoc test. Data are presented as mean ± SEM.

In accordance with the reduced endothelial function, we found a significant increase of (peri-) vascular fibrosis in the aortas of CD4-IL-17A^ind/+^ mice in comparison to control mice as shown by histological MTC-VES and Sirius red staining of aortic cross-sections (Figure 4(a), a1, a2, and a3). On the other hand, aortic wall thickness was not changed, as documented by MTC-VES staining (Figure 4(a), a1 and a3). To further examine the role of vascular fibrosis formation in vascular function, we analyzed the sprouting capacity of aortic rings but found no significant differences in length and number of sprouts after four days in culture (Figure 4(b)). However, the sprouts of CD4-IL-17A^ind/+^ mouse aortas contained significantly more fibroblasts (Figure 4(c)). In contrast, staining for endothelial cells and smooth muscle cells revealed no difference at baseline in fixed aortic sections of both groups of mice (Figure S3).

Next, we investigated the direct effect of IL-17A on mouse fibroblasts and vascular smooth muscle cells (VSMCs): isolated cells were incubated with increasing concentrations of IL-17A. We found that even low concentrations of IL-17A induced fibroblast proliferation whereas VSMC proliferation was not affected (Figure 4(d)). We conclude that chronic T cell-derived IL-17A exposition drives vascular fibrosis formation by fibroblast proliferation. This contributes to the phenotype of vascular dysfunction.

### 3.4. T Cell-Derived IL-17A Leads to Downregulation of Soluble Guanylate Cyclase Subunits *α*1 and *β*1

**Figure 5:**
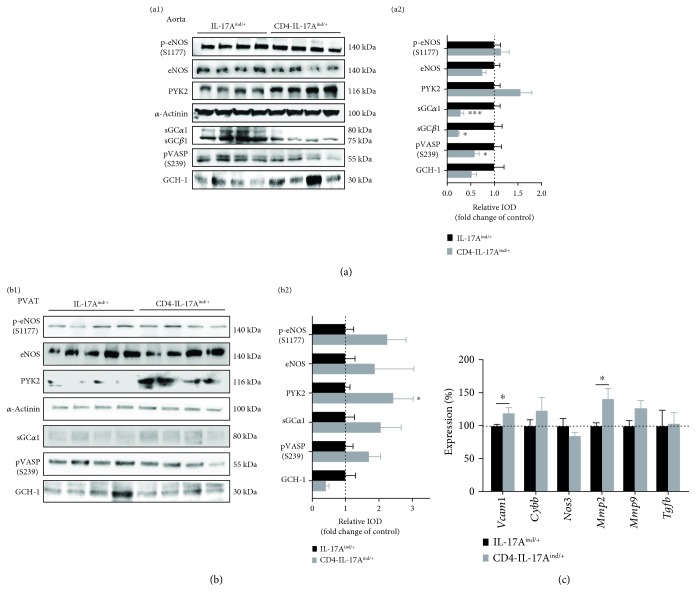
T cell-specific IL-17A overexpression results in a downregulation of sGC/VASP signaling in the aorta, with a concomitant upregulation of *Vcam1* and *Mmp2* mRNA. (a, b) Western blot of aortic protein (a) and PVAT protein (b) of CD4-IL-17A^ind/+^ and control mice of p-eNOS (S1177), eNOS, PYK2, sGC*α*1, sGC*β*1, pVASP (S239), and GCH-1. a1, b1: original Western blot; a2, b2: quantification of the signal, aorta: *n* = 4–16 (partially, 3-4 aortas were pooled per sample), Mann–Whitney *t*-test or unpaired Student's *t*-test. PVAT: *n* = 5–11, Student's unpaired *t*-test. (c) Quantitative real-time PCR of aortic mRNA of *Vcam1*, *Cybb*, *Nos3*, *Mmp2*, *Mmp9*, and *Tgfb*, *n* = 4–8, Mann–Whitney *t*-test or unpaired Student's *t*-test. Data are shown as mean ± SEM.

Being aware of an increased aortic stiffness due to IL-17A-mediated vascular fibrosis formation, we aimed to find out in what way vascular function signaling was altered in CD4-IL-17A^ind/+^ mice and consequently performed protein analysis of aortic tissue. We found that the soluble guanylate cyclase (sGC) subunits *α*1 and *β*1 were significantly downregulated in CD4-IL-17A^ind/+^ mice (Figure 5(a)). Furthermore, phosphorylation of vasodilator-stimulated phosphoprotein (pVASP), a target of the sGC/cGMP-dependent protein kinase G, was decreased (Figure 5(a)). We also identified that PYK2 was significantly upregulated in the PVAT of CD4-IL-17A^ind/+^ mice. When we analyzed the aortic mRNA expression, we found an upregulation of the profibrotic transcription marker matrix metalloproteinase 2 (*Mmp2*) and of the vascular cell adhesion molecule 1 (*Vcam1*).

In summary, chronic exposition to T cell-derived IL-17A led to a significant endothelial dysfunction. This was based on vascular fibrosis formation and on a downregulation of the sGC-cGMP pathway in the aorta more than on infiltration of myeloid cells into the vessel wall. Moreover, IL-17A increased the reactivity of myeloid cells, which resulted in higher peripheral ROS/RNS levels confronting the vessel wall with an aggressive blood milieu.

## 4. Discussion

Madhur et al. first showed that IL-17A is crucial in the development of AngII-induced vascular dysfunction and hypertension [4]. Moreover, treating mice with an IL-17A neutralizing antibody in parallel to four weeks of AngII infusion, led to a decreased aortic inflammation, lowered blood pressure, and reduced end organ damage in mice [5]. Due to the neutrophil-recruiting effect of IL-17A [9], we assumed that immune cell infiltration was the key mediator of vascular tone dysregulation in mice overexpressing IL-17A in T cells showing a severe vascular dysfunction that is comparable to that seen in AngII-treated mice [1]. In our mouse model, IL-17A was produced by CD4^+^ T cells which are in general one important source of IL-17A [8] and also by CD8^+^ T cells which usually express only very little IL-17A [31]. However, no significant increase of infiltrating immune cells in the aorta or in the PVAT was detectable among chronic T cell-derived IL-17A expositions. Instead, we noticed a significant increase of ROS/RNS production in the whole blood of CD4-IL-17A^ind/+^ mice, which was not accompanied by a systemic increase in myeloid cell numbers. Hence, we investigated the specific activity of primary isolated CD11b^+^ cells and found an increased ROS/RNS production of CD11b^+^ splenocytes in CD4-IL-17A^ind/+^ mice compared to controls in line with previous findings showing that IL-17A-dependent neutrophil activation induces a milieu disrupting the antioxidant homeostasis [32]. Our data demonstrate that T cell-derived IL-17A can disrupt the oxidant-antioxidant balance and induce oxidative stress leading to endothelial dysfunction.

An increased production of ROS/RNS can result in the uncoupling of the endothelial NO synthase (eNOS). eNOS and its downstream effectors sGC, cGMP, and cGMP-dependent protein kinase G (PKG) are the cornerstone of NO homeostasis and vascular tone regulation [33]. The complex crosstalk of signaling molecules that fine-tune the efficient NO synthesis is regulated by posttranslational modifications and protein-protein interactions, with eNOS phosphorylation at Ser1177 being the main activation site. In CD4-IL-17A^ind/+^ aortas, NO homeostasis as seen by eNOS phosphorylation at Ser1177, eNOS expression and uncoupling, was not altered. In addition, there was no change in aortic NO levels in CD4-IL-17A^ind/+^ mice compared to control mice. In contrast, Nguyen et al. had shown before that acute treatment of endothelial cells with IL-17A led to a phosphorylation of eNOS at its inhibitory site and a decrease of eNOS phosphorylation at Ser1177, resulting in an impaired NO homeostasis [17]. However, data on the effect of IL-17A on eNOS regulation are contradictory. One week of IL-17A infusion induced hypertension in mice, and this effect was attributed to the upregulation of Rho-A, a redox-related protein [17]. Mai et al. showed that genetic ablation of IL-17A in ApoE^−/−^ mice did not have an impact on either Ser1177 or Thr495 eNOS phosphorylation in vivo, but the observed mechanism was again redox- and inflammation-signaling relevant [34]. In our study, the effect of IL-17A on eNOS was mainly indirect and mediated by its oxidative stress and inflammation-inducing properties, but not accompanied by its uncoupling. Previous studies have shown the impact of mitochondrial Cyclophilin D (CypD) on ROS production and on the regulation of vascular function and hypertension [28, 35]. Proinflammatory cytokines such as IL-17A and TNF-*α* seemed to aggravate mitochondrial ROS production [35]. This exact effect might also play a role in our model of T cell-specific IL-17A overexpression, too.

Though, we observed a significant decrease in aortic soluble guanylate cyclase (sGC) subunits being accompanied by the reduced phosphorylation of VASP at Ser239, a surrogate marker of PKG [36]. sGC is important in NO signaling, commonly referred as the “NO receptor.” However, sGC is an enzyme which is highly sensitive to redox environment, as it is shown that elevated oxidative stress can change its redox state and lead to its deactivation [37]. It is known that sGC is affected by oxidation/loss of the sGC heme, oxidation, or nitrosation of cysteine residues and phosphorylation [38, 39]. These findings are consistent with our observations showing that increased oxidative stress levels, mediated through IL-17A and myeloid cell stimulation, resulted in an intravascular sGC downregulation. Notably though, it is the first time that a correlation between IL-17A and sGC has been established and can be attributed as a potent mechanism in the induction of vascular dysfunction.

Apart from the direct impairment of the cardiovascular system upon vast ROS/RNS formation, we identified changes also in ROS/RNS-related pathways that appear to contribute to the manifested vascular dysfunction seen in the mice that overexpress IL-17A in T cells. One of these pathways is the proline-rich tyrosine kinase 2 (PYK2) that is a member of the focal adhesion kinase (FAK) family and plays a pivotal role in a plethora of cellular events related with cell migration, proliferation, and survival [40]. Importantly, PYK2 can be activated upon ROS and cytokine release [41]. In the CD4-IL-17A^ind/+^ mice, we documented the upregulation of PYK2 in the PVAT. This could be a complemental finding to the redox-related mechanisms of IL-17A. It has been reported that adverse phosphorylation at Tyr657 by PYK2 represents a master switch of eNOS activity in AngII-induced hypertension [42] and may also explain the vascular dysfunction in the CD4-IL-17A^ind/+^ mice.

Our findings are in agreement to the observation that mice lacking the T cell-derived IL-17A are protected against aortic stiffening in response to chronic AngII infusion [19]. Furthermore, the association of T cell-driven IL-17A-mediated oxidative decay of the vasculature has been contemporarily described in a transgenic mouse model of a NADPH oxidase subunit p22 (phox) overexpression in smooth muscle cells [43]. The animals were shown to develop vascular collagen deposition, aortic stiffening, renal dysfunction, and hypertension with age and presented a significant overproduction of IL-17A and IFN-*γ* [43]. Here, we detected an increased fibroblast activity in aortic ring sprouting assay, where mainly fibroblasts were growing from isolated aortic rings of CD4-IL-17A^ind/+^ mice after four days in culture. Furthermore, our results clearly show the capacity of IL-17A to induce only fibroblast proliferation, but not VSMC proliferation in culture. This is in line with the increased collagen deposition in CD4-IL-17A^ind/+^ aortas without general thickening of the vessel wall. The profibrotic effect of IL-17A and elevated IL-17RA in human individuals with idiopathic pulmonary fibrosis and rheumatoid arthritis-associated lung disease has already been established [44]. In this pathogenicity, a direct effect of IL-17A/IL-17RA on the human fibroblastic population was attributed to the induction of NF*κ*B signaling and was successfully abrogated by pharmacological inhibitors and siRNA experiments [44]. Therefore, our results verify the direct crosstalk between IL-17A overexpression and fibrosis formation.

In conclusion, our data stress the essential role of IL-17A in the development of vascular dysfunction. We present that T cell-derived IL-17A leads to the induction of oxidative stress through activation of peripheral myeloid cells but does not result in an infiltration of myeloid cells in the vessel wall. IL-17A directly induces fibroblast proliferation leading to vascular collagen deposition. In parallel, elevated ROS/RNS downregulate the redox-sensitive soluble guanylate cyclase, reducing its activity and impairing NO signaling, independently of eNOS regulation and vascular immune cell infiltration.

## Supplementary Materials

Supplementary 1 Figure S1: CD4-IL-17A^ind/+^ mice show GFP expression in T cells. GFP signal in T cells in (A) the spleen and (B) aorta, representative plot of *n* = 7 mice per group. (A) Cells were gated on CD3^+^ (left), CD4^+^, and CD8^+^ cells (middle), and from all CD4^+^ cells, the GFP expression was analyzed. (B) Cells were pregated on CD90.2^+^ cells, and GFP expression was analyzed in this population.

Supplementary 2 Figure S2: No inflammatory infiltration in perivascular adipose tissue or kidneys. Flow cytometric analysis of (A) PVAT and (B) kidneys of CD4-IL-17A^ind/+^ and IL-17A^ind/+^ control mice. Gating strategy as described in Figure 2 (A); *n* = 3 (A) and 4‐12 (B); unpaired Student's *t*-test or Mann–Whitney *t*-test was performed. Data are shown as mean ± SEM.

Supplementary 3 Figure S3: No change in endothelial cells and vascular smooth muscle cells of CD4-IL 17A^ind/+^ aortas compared to controls. Aortic sections were stained for DAPI (blue), CD31 (red), and *α*-smooth muscle cell actin (*α*SMa, yellow); scale bar = 60 *μ*m. A representative image per group is shown, *n* = 3 mice per group.

Supplementary 4 Figure S4: Scheme of the influence of IL-17A on NO signaling. T cell-derived IL-17A stimulates myeloid cells to produce ROS/RNS. Increased levels of ROS/RNS induce the downregulation of sGC, which decreases the phosphorylation of VASP. Consequently, this causes an impaired vascular function. In parallel, ROS/RNS and IL-17A directly induce fibroblast proliferation leading to collagen deposition in the vessel wall, which impedes the vascular function, too.
