# Supplementary material for: T Cell-Derived IL-17A Induces Vascular Dysfunction via Perivascular Fibrosis Formation and Dysregulation of ·NO/cGMP Signaling
Source: Oxid Med Cell Longev. 2019 Jul 18;2019:6721531. doi: 10.1155/2019/6721531 (PMC6668561; doi:10.1155/2019/6721531)
Supplement: Supplementary 4 — Figure S4: Scheme of the influence of IL-17A on NO signaling. T cell-derived IL-17A stimulates myeloid cells to produce ROS/RNS. Increased levels of ROS/RNS induce the downregulation of sGC, which decreases the phosphorylation of VASP. Consequently, this causes an impaired vascular function. In parallel, ROS/RNS and IL-17A directly induce fibroblast proliferation leading to collagen deposition in the vessel wall, which impedes the vascular function, too. [file 6721531.f4.pptx]

## Slide 1
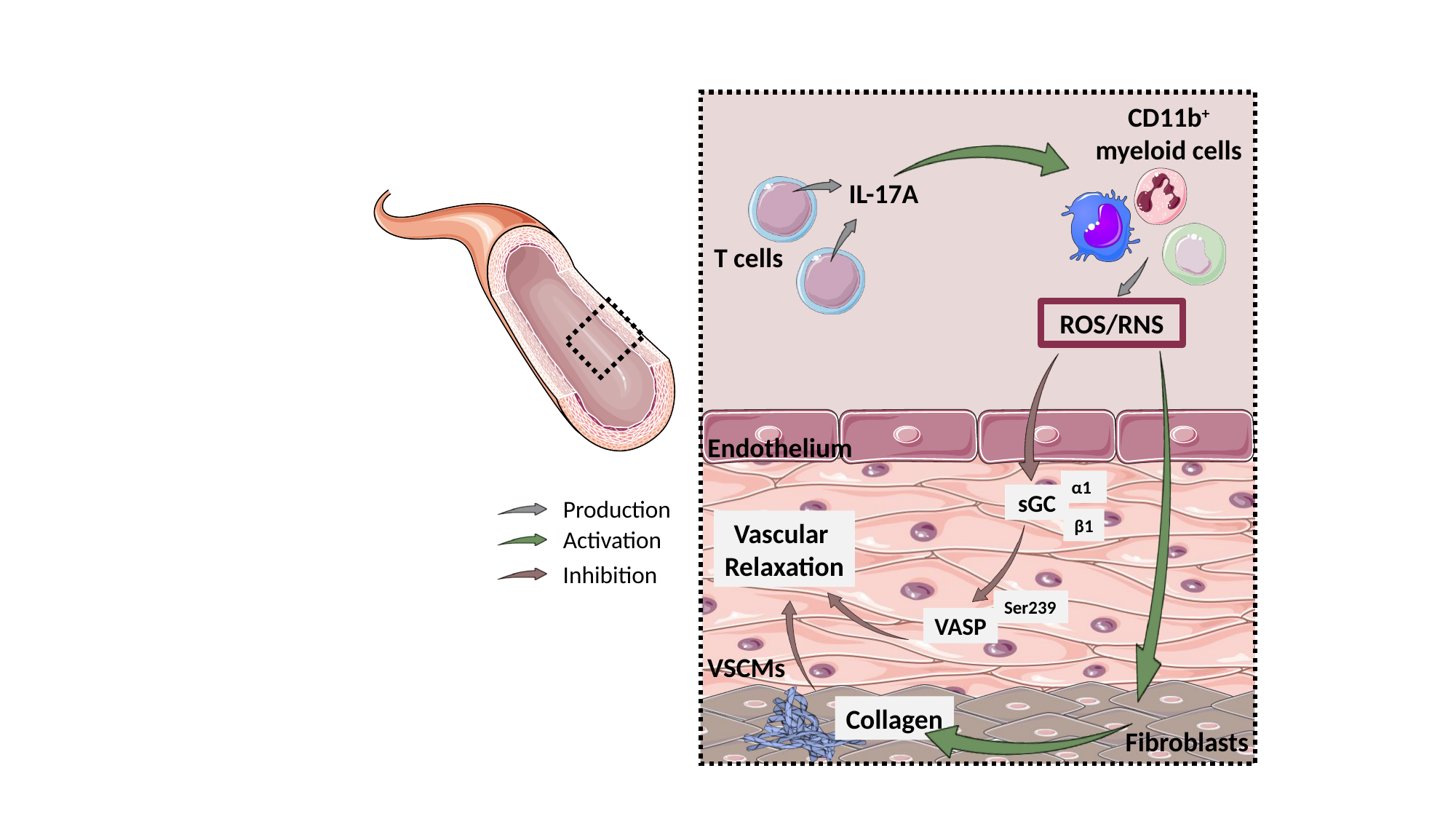

CD11b+ myeloid cells
IL-17A
T cells
ROS/RNS
Endothelium
α1
sGC
Production
β1
Vascular
Relaxation
Activation
Inhibition
Ser239
VASP
VSCMs
Collagen
Fibroblasts
